# Inclusions in a Single Variable in Ultrametric Spaces and Hyers-Ulam Stability

**DOI:** 10.1155/2013/129637

**Published:** 2013-10-01

**Authors:** Magdalena Piszczek

**Affiliations:** Pedagogical University, Podchorążych 2, 30-084 Kraków, Poland

## Abstract

We present some properties of set-valued inclusions in a single variable in ultrametric spaces. As a consequence, we obtain stability results for the corresponding functional equations.

## 1. Introduction

 A metric space (*X*, *d*) is called an ultrametric space (or non-Archimedean metric space), if *d*, called an ultrametric, satisfies the strong triangle inequality

(1) $$\begin{array}{c} d\left(x,z\right)\leq \max\left\{d\left(x,y\right),d\left(y,z\right)\right\}\;\text{for}\;x,y,z\in X. \end{array}$$

One of the typical ultrametrics is a *p*-adic metric. Let *p* be a fixed prime. For *n*, *m* ∈ *ℕ*, we define

(2) $$\begin{array}{c} d\left(m,n\right)=\{\begin{array}{cc} 0 & \text{if}\;m=n,\\ p^{-r} & \text{if}\;m\neq n, \end{array} \end{array}$$

where *r* is the largest nonnegative integer such that *p* ^*r*^ divides *m* − *n*. This example is the introduction to the *p*-adic numbers which play the essential role because of their connections with some problem coming from quantum physics, *p*-adic string or superstring (see [1]).

The inequality

(3) d(xm,xn)≤max⁡{d(xm,xm−1),…,d(xn+1,xn)}for  m>n

yields and implies the following lemma.

**Lemma 1**

A sequence (*x*_*n*_)_*n*∈  *ℕ*_ in an ultrametric space (*X*, *d*) is a Cauchy sequence if and only if lim⁡_*n*→*∞*_ *d*(*x*_*n*+1_, *x*_*n*_) = 0.

Let (*X*, *d*) be an ultrametric space. The number *δ*(*A*) = sup⁡{*d*(*x*, *y*) : *x*, *y* ∈ *A*} is called the diameter of *A* ⊂ *X*. We will denote by *n*(*X*) the family of all nonempty subsets of *X*. Moreover, let *bd*(*X*) stand for the family of all bounded sets of *n*(*X*) let and *b*cl⁡(*X*) denote the family of all closed sets of *bd*(*X*). We understand the convergence of sets with respect to the Hausdorff metric *h* derived from the metric *d*. It is easy to see that (*b*cl⁡(*X*), *h*) is also an ultrametric space, that is, *h* satisfies the strong triangle inequality

(4) $$\begin{array}{c} h\left(A,C\right)\leq \max\left\{h\left(A,B\right),h\left(B,C\right)\right\}\;\text{for}\;A,B,C\in b\mathrm{cl}\left(X\right). \end{array}$$

We say that (*X*, +,  *d*) is a complete ultrametric commutative groupoid with 0, if (*X*, +) is a commutative groupoid with a neutral element 0, (*X*, *d*) is a complete ultrametric space and the operation + is continuous with respect to the metric *d*.

From now on, we assume that *K* is a nonempty set and (*X*, +, *d*) is a complete ultrametric commutative groupoid with 0. For *A*, *B* ∈ *n*(*X*), we define

(5) $$\begin{array}{c} A+B=\left\{a+b:a\in A,b\in B\right\}. \end{array}$$

The aim of the paper is to obtain some results concerning the following inclusion:

(6) $$\begin{array}{c} \Psi \left(F\left(a\left(x\right)\right)\right)\subset F\left(x\right)+G\left(x\right), \end{array}$$

where Ψ : *X* → *X*, *F*, *G* : *K* → *n*(*X*), and *a* : *K* → *K* and its generalization in an ultrametric space. In ultrametric spaces, it is possible to get better estimation with weaker assumptions, than in metric spaces. The ideas of proofs are based on the ideas in [2]. As a consequence we obtain stability results for the corresponding functional equation Ψ∘*f*∘*a* = *f* and its generalization. Some results of the stability of functional equations in non-Archimedean spaces can be found in [3–8].

## 2. Main Results

**Theorem 2**

 Let *F*, *G* : *K* → *n*(*X*), 0 ∈ *G*(*x*) for all *x* ∈ *K*, Ψ : *X* → *X*, *a* : *K* → *K*, *λ* ∈ (0,1), and

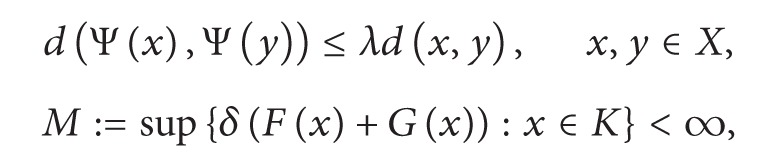
(7)

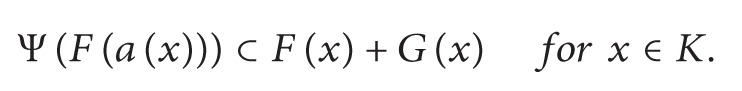
(8)
Then there exists a unique function *f* : *K* → *X* such that Ψ∘*f*∘*a* = *f* and

(9) $$\begin{array}{c} d\left(f\left(x\right),F\left(x\right)\right)\leq M,\;x\in K. \end{array}$$

**Proof**

Let us fix *x* ∈ *K*. Replacing *x* by *a* ^*n*^(*x*) in (8), we get

(10) $$\begin{array}{c} \Psi \left(F\left(a^{n+1}\left(x\right)\right)\right)\subset F\left(a^{n}\left(x\right)\right)+G\left(a^{n}\left(x\right)\right), \end{array}$$

and as 0 ∈ *G*(*x*), we have

(11) $$\begin{array}{c} F\left(a^{n}\left(x\right)\right)\subset F\left(a^{n}\left(x\right)\right)+G\left(a^{n}\left(x\right)\right) \end{array}$$

for *n* ∈ *ℕ*_0_. Thus,

(12) h(Ψn+1(F(an+1(x))),Ψn(F(an(x))))  ≤λnh(Ψ(F(an+1(x))),F(an(x)))  ≤λnδ(F(an(x))+G(an(x)))≤λnM

for all *n* ∈ *ℕ*_0_. According to Lemma 1 and lim⁡_*n*→*∞*_ *λ* ^*n*^ *M* = 0, the sequence (Ψ^*n*^(*F*(*a* ^*n*^(*x*))))_*n*∈*ℕ*_0__ is a Cauchy sequence. Since (*b*cl⁡(*Y*), *h*) is complete, there exists the limit lim⁡_*n*→*∞*_cl⁡Ψ^*n*^(*F*(*a* ^*n*^(*x*))). Moreover,

(13) $$\begin{array}{c} \delta \left(\mathrm{cl}\Psi^{n}\left(F\left(a^{n}\left(x\right)\right)\right)\right)\leq \lambda^{n}\delta \left(F\left(a^{n}\left(x\right)\right)\right), \end{array}$$

and the right side of the last inequality converges to 0 with *n* → *∞*. Therefore,

(14) $$\begin{array}{c} \underset{n\to \infty }{\lim}\mathrm{cl}\Psi^{n}\left(F\left(a^{n}\left(x\right)\right)\right)=:f\left(x\right) \end{array}$$

is a singleton and as Ψ is continuous,

(15) Ψ(f(a(x)))  =Ψ(lim⁡n→∞cl⁡Ψn(F(an(a(x)))))  ⊂lim⁡n→∞cl⁡Ψn+1(F(an+1(x)))=f(x),

so Ψ∘*f*∘*a* = *f*. Notice that

(16) h(Ψn(F(an(x))),F(x))  ≤max⁡⁡{h(Ψn(F(an(x))),Ψn−1(F(an−1(x)))),…,     h(Ψ(F(a(x))),F(x))}  ≤max⁡⁡{λn−1δ(F(an−1(x))+G(an−1(x))),…,     δ(F(x)+G(x))}  ≤max⁡{λn−1M,…,M}≤M.

Consequently,

(17) $$\begin{array}{c} d\left(f\left(x\right),F\left(x\right)\right)\leq M,\\ f\left(x\right)\in F\left(x\right)+MS, \end{array}$$

where *S* is a closed unit ball.

It remains to prove the uniqueness of *f*. Let *f*, *g* be such that Ψ∘*f*∘*a* = *f*, Ψ∘*g*∘*a* = *g*, *d*(*f*(*x*), *F*(*x*)) ≤ *M*, *d*(*g*(*x*), *F*(*x*)) ≤ *M* for *x* ∈ *K*. By induction we get Ψ^*n*^∘*f*∘*a* ^*n*^ = *f* and Ψ^*n*^∘*g*∘*a* ^*n*^ = *g* for *n* ∈ *ℕ*_0_. Hence,

(18) d(f(x),g(x))=d(Ψn∘f∘an(x),Ψn∘g∘an(x))≤λnd(f(an(x)),g(an(x)))≤λnδ(F(an(x))+MS), n∈ℕ0.

Since lim⁡_*n*→*∞*_ *λ* ^*n*^ *δ*(*F*(*a* ^*n*^(*x*)) + *MS*) = 0, we have *f* = *g*.

**Theorem 3**

Assume that *F*, *G* : *K* → *bd*(*X*), 0 ∈ *G*(*x*) for all *x* ∈ *K*, *k* ∈ *ℕ*, Ψ : *K* × *X* ^*k*^ → *X*, *α*_1_,…, *α*_*k*_ : *K* → *K*, *λ*_1_,…, *λ*_*k*_ : *K* → [0, *∞*) are such that

(19) d(Ψ(x,w1,…,wk),Ψ(x,z1,…,zk))  ≤max⁡i∈{1,…,k}λi(x)d(wi,zi)

for *x* ∈ *K*, *w*_1_,…, *w*_*k*_, *z*_1_,…, *z*_*k*_ ∈ *X*,

(20) lim⁡n→∞ max⁡i1=1,…,kλi1(x)max⁡i2=1,…,k(λi2∘αi1)(x)⋯  max⁡in=1,…,k(λin∘αin−1∘⋯∘αi1)(x)   ×δ(F((αin∘⋯∘αi1)(x))+ G((αin∘⋯∘αi1)(x)))=0, x∈K,

(21) Ψ(x,F(α1(x)),…,F(αk(x)))  ⊂F(x)+G(x) for  x∈K.

Then there exists a unique function *f* : *K* → *X* such that Ψ(*x*, *f*(*α*_1_(*x*)),…, *f*(*α*_*k*_(*x*))) = *f*(*x*) and *d*(*f*(*x*), *F*(*x*)) ≤ *k*(*x*) for *x* ∈ *K*, where

(22) k(x)=sup⁡n∈ℕ{δ(F(x)+G(x)),max⁡i1=1,…,kλi1(x)max⁡i2=1,…,k(λi2∘αi1)(x)⋯max⁡in=1,…,k(λin∘αin−1∘⋯∘αi1)(x) ×δ(F((αin∘⋯∘αi1)(x)) +G((αin∘⋯∘αi1)(x)))}.

**Proof**

Let us fix *x* ∈ *K*. Replacing *x* by *α*_*i*_(*x*), *i* = 1,…, *k*, in (21), we obtain

(23) Ψ(αi(x),F(α1(αi(x))),…,F(αk(αi(x))))  ⊂F(αi(x))+G(αi(x)).

Since 0 ∈ *G*(*x*), we have *F*(*α*_*i*_(*x*)) ⊂ *F*(*α*_*i*_(*x*)) + *G*(*α*_*i*_(*x*)) for *i* = 1,…, *k*. Hence,

(24) Ψ(x,Ψ(α1(x),F(α1(α1(x))),…,F(αk(α1(x)))),…,Ψ(αk(x),F(α1(αk(x))),…,F(αk(αk(x)))))    ⊂Ψ(x,F(α1(x))+G(α1(x)),…,F(αk(x))+ G(αk(x))).

We define a sequence (*A*_*n*_(*x*))_*n*∈*ℕ*_0__ by the following recurrence relation:

(25) A0(x)=F(x),An+1(x)=Ψ(x,An(α1(x)),…,An(αk(x))).

It is easy to see that

(26) h(A1(x),A0(x))  ≤h(Ψ(x,F(α1(x)),…,F(αk(x))),F(x))  ≤δ(F(x)+G(x)),h(An+1(x),An(x))  ≤max⁡i1=1,…,kλi1(x)max⁡i2=1,…,k(λi2∘αi1)(x)⋯   max⁡in=1,…,k(λin∘αin−1∘⋯∘αi1)(x)     ×δ(F((αin∘⋯∘αi1)(x))+G((αin∘⋯∘αi1)(x)))

for *n* ∈ *ℕ*. In virtue of (20), the sequence (*A*_*n*_(*x*))_*n*∈*ℕ*_ is a Cauchy sequence. As (*b*cl⁡(*Y*), *h*) is a complete metric space, there exists the limit lim⁡_*n*→*∞*_cl⁡*A*_*n*_(*x*). Moreover,

(27) δ(cl⁡An(x))  ≤max⁡i1=1,…,kλi1(x)max⁡i2=1,…,k(λi2∘αi1)(x)⋯   max⁡in=1,…,k(λin∘αil−1∘⋯∘αi1)(x)     ×δ(F((αin∘⋯∘αi1)(x))),

and the right side of the last inequality converges to 0 as *n* → *∞*. Therefore,

(28) $$\begin{array}{c} \underset{n\to \infty }{\lim}\mathrm{cl}A_{n}\left(x\right)=:f\left(x\right) \end{array}$$

is a singleton, and as Ψ satisfies (19),

(29) Ψ(x,f(α1(x)),…,f(αk(x)))  =Ψ(x,lim⁡n→∞cl⁡An(α1(x)),…,lim⁡n→∞cl⁡An(αk(x)))   ⊂lim⁡n→∞cl⁡Ψ(x,An(α1(x)),…,An(αk(x)))  =lim⁡n→∞cl⁡An+1(x)=f(x).

By (26), we get

(30) h(An(x),F(x))  ≤max⁡{δ(F(x)+G(x)),…,      max⁡i1=1,…,kλi1(x)max⁡i2=1,…,k(λi2∘αi1)(x)⋯      max⁡in−1=1,…,k(λin−1∘αin−2∘⋯∘αi1)(x)      ×δ(F((αin−1∘⋯∘αi1)(x))   +G((αin−1∘⋯∘αi1)(x)))}

which, with *n* → *∞*, yields

(31) d(f(x),F(x))≤sup⁡n∈ℕ  {δ(F(x)+G(x)),     max⁡i1=1,…,kλi1(x)max⁡i2=1,…,k(λi2∘αi1)(x)⋯   max⁡in=1,…,k(λin∘αin−1∘⋯∘αi1)(x)   ×δ(F((αin∘⋯∘αi1)(x))       + G((αin∘⋯∘αi1)(x)))}=k(x).

It remains to prove the uniqueness of *f*. Suppose that *f*, *g* are such that Ψ(*x*, *f*(*α*_1_(*x*)),…, *f*(*α*_*k*_(*x*))) = *f*(*x*), Ψ(*x*, *g*(*α*_1_(*x*)),…, *g*(*α*_*k*_(*x*))) = *g*(*x*), *d*(*f*(*x*), *F*(*x*)) ≤ *k*(*x*), and *d*(*g*(*x*), *F*(*x*)) ≤ *k*(*x*). Replacing *x* by *α*_*i*_(*x*), *i* = 1,…, *k*, in the penultimate equality, we get

(32) $$\begin{array}{c} \Psi \left(\alpha_{i}\left(x\right),f\left(\alpha_{1}\left(\alpha_{i}\left(x\right)\right)\right),\ldots ,f\left(\alpha_{k}\left(\alpha_{i}\left(x\right)\right)\right)\right)=f\left(\alpha_{i}\left(x\right)\right). \end{array}$$

Thus,

(33) Ψ(x,Ψ(α1(x),f(α1(α1(x))),…,f(αk(α1(x)))),…,  Ψ(αk(x),f(α1(αk(x))),…,f(αk(αk(x)))))    =Ψ(x,f(α1(x)),…,f(αk(x)))=f(x),

and we get a constant sequence

(34) f0(x)=f(x),fn+1(x)=Ψ(x,fn(α1(x)),…,fn(αk(x))).

In the same way, we get a constant sequence

(35) $$\begin{array}{c} g_{0}\left(x\right)=g\left(x\right),\\ g_{n+1}\left(x\right)=\Psi \left(x,g_{n}\left(\alpha_{1}\left(x\right)\right),\ldots ,g_{n}\left(\alpha_{k}\left(x\right)\right)\right). \end{array}$$

Hence,

(36) d(f(x),g(x))=d(fn(x),gn(x))≤max⁡i1=1,…,kλi1(x)max⁡i2=1,…,k(λi2∘αi1)(x)⋯ max⁡in=1,…,k(λin∘αin−1∘⋯∘αi1)(x) ×d(f((αin∘⋯∘αi1)(x)),g((αin∘⋯∘αi1)(x)))≤max⁡i1=1,…,kλi1(x)max⁡i2=1,…,k(λi2∘αi1)(x)⋯ max⁡in=1,…,k(λin∘αin−1∘⋯∘αi1)(x) ×(d(f((αin∘⋯∘αi1)(x)),F((αin∘⋯∘αi1)(x)))+d(F((αin∘⋯∘αi1)(x)),g((αin∘⋯∘αi1)(x))))≤2max⁡i1=1,…,kλi1(x)max⁡i2=1,…,k(λi2∘αi1)(x)⋯ max⁡in=1,…,k(λin∘αin−1∘⋯∘αi1)(x) ×k((αin∘⋯∘αi1)(x)).

Using the definition of *k*, we get

(37) d(f(x),g(x))≤2max⁡i1=1,…,kλi1(x)max⁡i2=1,…,k(λi2∘αi1)(x)⋯  max⁡in=1,…,k(λin∘αin−1∘⋯∘αi1)(x) ×(δ(F((αin∘⋯∘αi1)(x))+G((αin∘⋯∘αi1)(x))+sup⁡l=1,…,∞ max⁡j1=1,…,k(λj1∘αin−1∘⋯∘αi1)×(x)⋯max⁡jl=1,…,k(λjl∘αjl−1∘⋯∘αj1∘αin−1∘⋯∘αi1)(x)×δ(F((αjl∘⋯∘αj1∘αin−1∘⋯∘αi1)(x))+G((αjn∘⋯∘αj1∘αin−1∘⋯∘αi1)(x)))))=sup⁡l=n,…,∞ max⁡ i1=1,…,kλi1(x)max⁡i2=1,…,k(λi2∘αi1)   ×(x)⋯max⁡il=1,…,k(λil∘αil−1∘⋯∘αi1)(x) ×δ(F((αil∘⋯∘αi1(x)))+ G((αil∘⋯∘αi1)(x))).

It follows that *f* = *g* with *n* → 0, and the proof is completed.

## 3. Stability Results

 We present the applications of the above theorems to the results concerning the stability of functional equations.

**Corollary 4**

 Let *f* : *K* → *X*, Ψ : *X* → *X*, *a* : *K* → *K*, *λ* ∈ (0,1), *ϵ* > 0 satisfy

(38) $$\begin{array}{c} d\left(\Psi \left(x\right),\Psi \left(y\right)\right)\leq \lambda d\left(x,y\right),\;x,y\in X,\\ d\left(\Psi \left(f\left(a\left(x\right)\right)\right),f\left(x\right)\right)\leq \epsilon ,\;x\in K. \end{array}$$

Then there exists a unique function *g* : *K* → *X* such that Ψ∘*g*∘*a* = *g* and

(39) $$\begin{array}{c} d\left(f\left(x\right),g\left(x\right)\right)\leq 2\epsilon ,\;x\in K. \end{array}$$

**Proof**

Let *F*(*x*) = {*f*(*x*)} for *x* ∈ *K*. Then

(40) $$\begin{array}{c} \Psi \left(F\left(a\left(x\right)\right)\right)\subset F\left(x\right)+\epsilon S, \end{array}$$

where *S* is a closed unit ball and

(41) $$\begin{array}{c} \delta \left(F\left(x\right)+\epsilon S\right)=2\epsilon . \end{array}$$

According to Theorem 2 there exists a unique function *g* such that Ψ∘*g*∘*a* = *g* and *d*(*f*(*x*), *g*(*x*)) ≤ 2*ϵ* for *x* ∈ *K*.

**Corollary 5**

Let *f* : *K* → *X*, *k* ∈ *ℕ*, *α*_1_,…, *α*_*k*_ : *K* → *K*, *λ*_1_,…, *λ*_*k*_ : *K* → [0,1], *ϵ* > 0, Ψ : *K* × *X* ^*k*^ → *X* satisfy (19) and

(42) $$\begin{array}{c} d\left(\Psi \left(x,f\left(\alpha_{1}\left(x\right)\right),\ldots ,f\left(\alpha_{k}\left(x\right)\right)\right),f\left(x\right)\right)\leq \epsilon \;for\;\;x\in K. \end{array}$$

Then there exists a unique function *g* such that

(43) $$\begin{array}{c} \Psi \left(x,g\left(\alpha_{1}\left(x\right)\right),\ldots ,g\left(\alpha_{k}\left(x\right)\right)\right)=g\left(x\right),\\ d\left(f\left(x\right),g\left(x\right)\right)\leq 2\epsilon . \end{array}$$

**Proof**

Let *F*(*x*) = {*f*(*x*)} for *x* ∈ *K*. Then

(44) $$\begin{array}{c} \Psi \left(x,F\left(\alpha_{1}\left(x\right)\right),\ldots ,F\left(\alpha_{k}\left(x\right)\right)\right)\subset F\left(x\right)+\epsilon S, \end{array}$$

where *S* is a closed unit ball and

(45) sup⁡l=1,…,∞{δ(F(x)+ϵS),max⁡i1=1,…,kλi1(x)max⁡i2=1,…,k(λi2∘αi1)(x)⋯    max⁡il=1,…,k(λil∘αil−1∘⋯∘αi1)(x)    × δ(F((αil∘…∘αi1)(x))+ϵS)}=2ϵ.

By Theorem 3, we get the assertion.

As it was observed in [9, 10] from the stability results concerning the equation Ψ∘*f*∘*a* = *f*, we can easily derive the stability of functional equations in several variables, for example, the Cauchy equation, the Jensen equation, or the quadratic equation. The equation Ψ(*x*, *f*(*α*_1_(*x*)),…, *f*(*α*_*k*_(*x*))) = *f*(*x*) is a generalization of the gamma-type equations or the linear equations (see [11, 12]).
